# Expression pattern of Stomatin-domain proteins in the peripheral olfactory system

**DOI:** 10.1038/s41598-022-15572-1

**Published:** 2022-07-06

**Authors:** Kevin Y. Gonzalez-Velandia, Andres Hernandez-Clavijo, Anna Menini, Michele Dibattista, Simone Pifferi

**Affiliations:** 1 Neurobiology Group, SISSA, Scuola Internazionale Superiore di Studi Avanzati, 34136 Trieste, Italy; 2 Department of Basic Medical Sciences, Neuroscience and Sensory Organs, University of Bari A. Moro, 70124 Bari, Italy; 3 Department of Experimental and Clinical Medicine, Università Politecnica delle Marche, 60126 Ancona, Italy

**Keywords:** Neuroscience, Cellular neuroscience, Olfactory system

## Abstract

Recent data show that Stomatin-like protein 3 (STOML3), a member of the stomatin-domain family, is expressed in the olfactory sensory neurons (OSNs) where it modulates both spontaneous and evoked action potential firing. The protein family is constituted by other 4 members (besides STOML3): STOM, STOML1, STOML2 and podocin. Interestingly, STOML3 with STOM and STOML1 are expressed in other peripheral sensory neurons: dorsal root ganglia. In here, they functionally interact and modulate the activity of the mechanosensitive Piezo channels and members of the ASIC family. Therefore, we investigated whether STOM and STOML1 are expressed together with STOML3 in the OSNs and whether they could interact. We found that all three are indeed expressed in ONSs, although STOML1 at very low level. STOM and STOML3 share a similar expression pattern and STOML3 is necessary for STOM to properly localize to OSN cilia. In addition, we extended our investigation to podocin and STOML2, and while the former is not expressed in the olfactory system, the latter showed a peculiar expression pattern in multiple cell types. In summary, we provided a first complete description of stomatin-domain protein family in the olfactory system, highlighting the precise compartmentalization, possible interactions and, finally, their functional implications.

## Introduction

Odor perception starts in the olfactory neuroepithelium with odorant molecules binding to receptors in olfactory sensory neurons (OSNs). OSNs are bipolar neurons that extend a dendrite with several apical cilia to the surface of the olfactory epithelium (OE) and an axon to the olfactory bulb (OB). Odorant molecules bind to odorant receptors in the cilia and initiate the transduction cascade that produces action potential firing that is transmitted to the OB for further processing (reviewed by^1–6^). In the olfactory system, STOML3 (originally named SRO or SLP3) is known to be expressed in OSNs, where it mainly localizes to the knob and proximal cilia^7–11^.

STOML3 is a member of the stomatin-domain family of integral membrane proteins that, in mammals, include stomatin (STOM), three stomatin-like (STOML) proteins STOML1, STOML2, STOML3 and podocin^12^. All members of the stomatin family are characterized by a conserved core domain called stomatin-domain^13,14^. The insertion in the membrane is due to a short hydrophobic hairpin located at the N-terminus of the protein^12^.

STOML3 was first found in OSNs^9^. It is known to be expressed in a limited number of tissues, including mechanosensory neurons of dorsal root ganglia (DRG), and to regulate Piezo and ASIC channels^15–18^.

STOM was the first identified member of the stomatin-domain protein family in mammals and it has been named from the hereditary human haemolytic anaemia, stomatocytosis^19^. Although stomatocytosis is not due to mutations of *STOM* gene, STOM is missing in red blood cells from stomatocytosis patients^20^. STOM is lost also in the stomatin-deficient cryohydrocytosis (sdCHC). sdCHC is a very rare and severe condition due to the mutation of glucose transporter GLUT1 encoded by *SLC2A1* gene^21^. Recent studies showed that stomatocytosis is a complex disease showing different phenotypes due to mutations in several genes^22^. Interestingly, also mutation in *Piezo1* causes stomatocytosis^23,24^. STOM is also express in DRG neurons^19,25^ and it modulates the skin mechanoreception possibly by the modulation of Piezo channel^26,27^.

STOML1 is highly expressed in the brain and at a lower level in the heart, skeletal muscle and DRG sensory neurons, where it modulates the activity of ASIC channels^27^. STOML2 is expressed in brain, heart, skeletal muscle, red blood cell and T cells^28,29^. Interestingly, STOML2 has been found in mitochondria where it binds cardiolipin stabilizing the inner mitochondrial membrane and creating a scaffold for supramolecular complex^30–33^. However, STOML2 is also localized on the plasma membrane and it could contribute to organize microdomains in immunological synapses of T cells^34^. The structural motive responsible for STOML2 membrane localization is still unknown since it does not have a hydrophobic membrane anchor at its N-terminus. Finally, STOML2 is overexpressed and associated with poor prognosis in many types of cancer^35–37^. Podocin is specifically localized to slit diaphragm of podocytes, a highly specialized epithelium covering the kidney glomerular capillary wall^38^. Mutation in *Podocin* causes the familial idiopathic nephrotic syndrome^39^.

Proteomics and transcriptomics data have shown that STOM, STOML1, STOML2 and STOML3 are expressed in the olfactory epithelium, but a precise localization of all stomatin-domain proteins in the olfactory system is still missing^40–46^. Here, we explored by immunohistochemistry the expression of the stomatin-domain proteins in the OE and OB and found that the four members of the family (but not podocin) are expressed in the olfactory system in different cellular and subcellular locations, where they are likely to play different roles.

## Results

### RT-PCR

**Figure 1:**
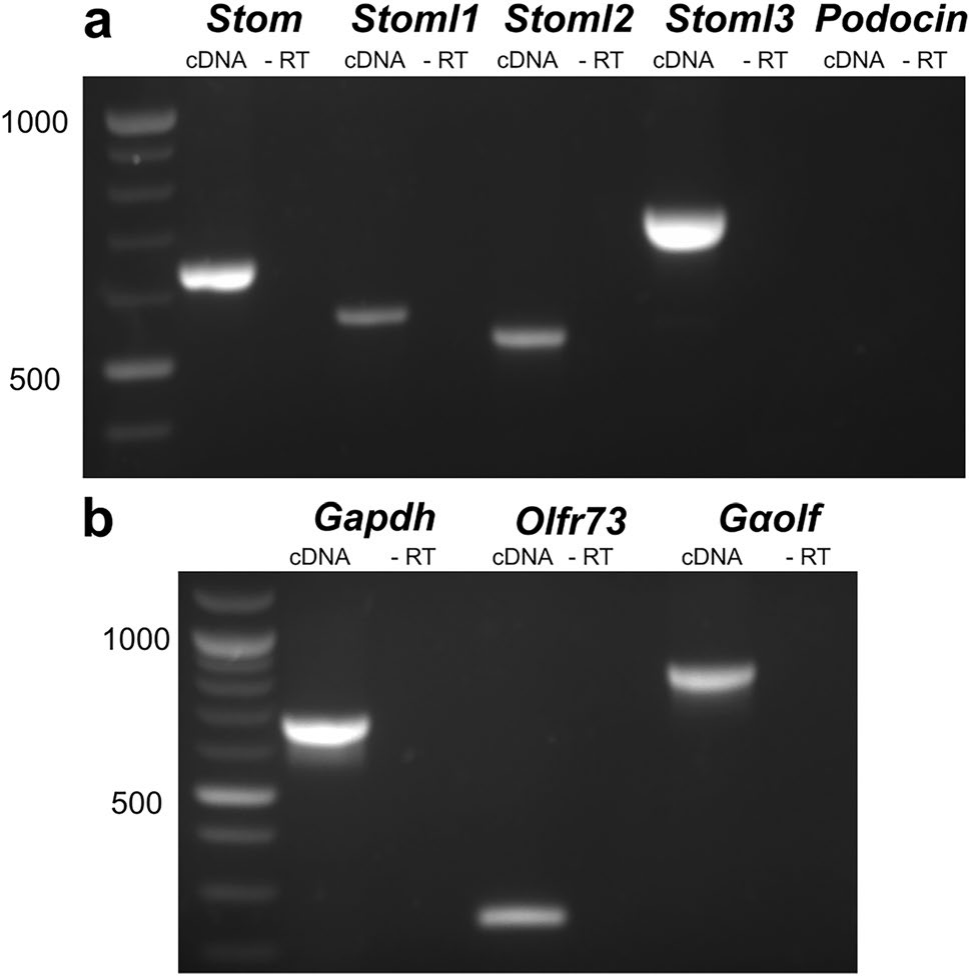
mRNAs from the stomatin-domain protein family expressed in the OE. Total mRNA extracted from the OE was amplified by RT-PCR using specific primers for *Stom, Stoml1, Stoml2, Stoml3*, and *Podocin* (**a**) To test the quality of the cDNA, RT-PCR amplifications were performed on *Gapdh, Olfr73, Gα*_*olf*_, (**b**) Control experiments were done for each target using the same sample template without retro-transcriptase.

The family of stomatin-domain proteins consists of five members: STOM, STOML1, STOML2, STOML3 and Podocin. To explore their expression in the olfactory epithelium, we used RT-PCR and detected mRNA for 4 out of 5 members: *Stom, Stoml1, Stoml2, Stoml3* (Fig. 1a)*.* We performed RT-PCR from the whole OE that was enriched in mRNA from OSNs, since we could detect mRNA of the olfactory receptor *Olfr73* and the mRNA encoding for a protein of the odorant signal transduction machinery, *Gα*_*olf*_ (Fig. 1b). A limitation of RT-PCR is that it was performed on the whole epithelium mRNA, thus not giving any information about the different cell types expressing stomatin-domain proteins.

To investigate the cellular localization of stomatin-domain proteins in the olfactory system, we performed localization and cell specific expression studies by using immunofluorescence techniques. We used commercially available antibodies and first tested their specificity on HEK-293 cells transiently transfected with plasmids containing the cDNA sequence of *Stom, Stoml1, Stoml2* or *Stoml3* fused with EGFP or mCherry. All the used antibodies were highly selective for their target protein (Suppl. Figure 1), making them a powerful tool for immunostaining analysis of the four stomatin-domain proteins in the olfactory system.

### STOM

**Figure 2:**
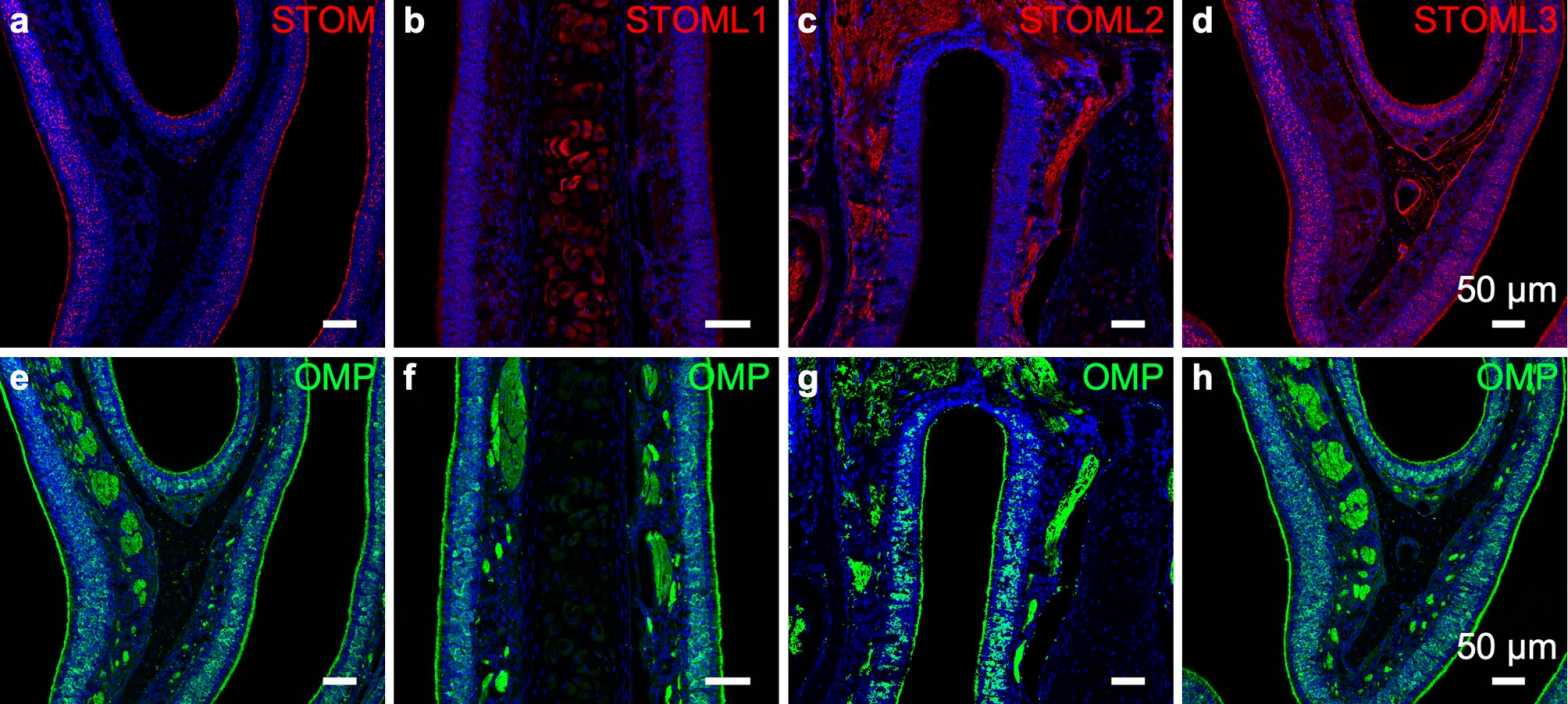
Stomatin-domain protein localization in olfactory areas of the nasal cavity. Confocal micrographs at low magnification of coronal sections of the OE of WT mice immuno-stained with antibodies (red) against STOM (**a**), STOML1 (**b**), STOML2 (**c**), and STOML3 (**d**). The same sections were double-stained with anti-OMP antibodies (**e**–**h**, green). At low magnification, STOML2 is mainly visible around the axon bundles of the OSNs, while STOML1 is detected in the cartilage of the nasal septum. STOM and STOML3 predominantly localize in the apical part of the OE along the interface with the lumen of the nasal cavity. Nuclei were stained with DAPI (blue).

**Figure 3:**
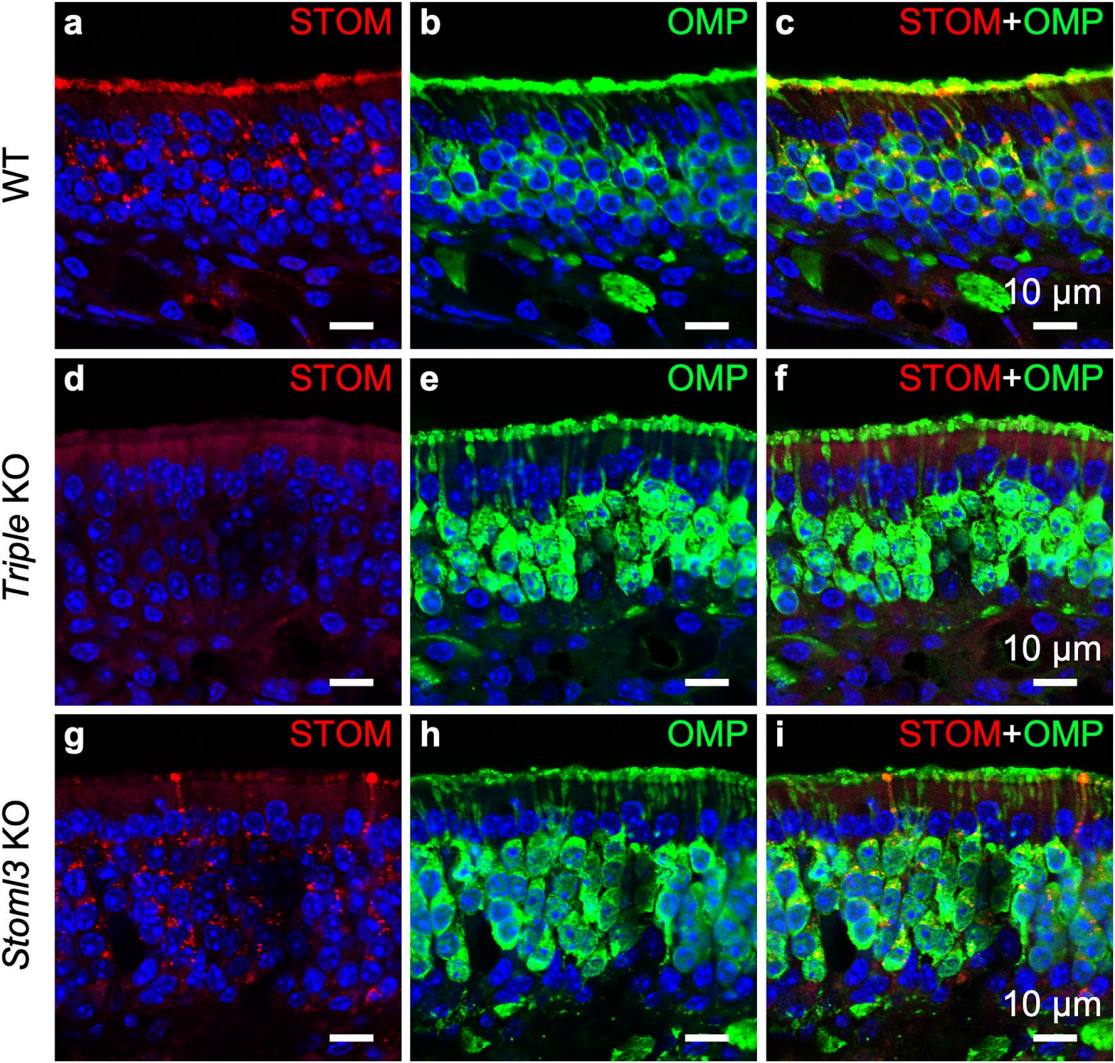
STOM expression in the olfactory epithelium. Confocal micrographs at high magnification of coronal sections of the olfactory epithelium of WT (**a**–**c**), *Triple* KO (**d**–**f**), and *Stoml3* KO (**g**–**i**) mice, double-stained with anti-STOM (red) and anti-OMP (green) antibodies. In WT mice, STOM mainly localizes in the knob/ciliary region of the OSNs while in the cell body it is detected in puncta (**a**–**c**). STOM staining is not present in *triple* KO mice, confirming the specificity of the signal observed in WT (**d**–**f**). In *Stoml3* KO mice, STOM is absent in the ciliary layer, and in some OSNs it remains trapped in their dendrites and knobs (**g**–**i**). Nuclei were stained with DAPI (blue).

Our immunofluorescence experiments revealed that STOM is expressed in the OE (Fig. 2a and e) and it is particularly enriched in the apical layer (Fig. 3a). This region contains the knobs, the terminal part of the OSN’s dendrites, and the cilia. Interestingly, several puncta were also present in the middle portion of the OE. By using antibodies raised against the olfactory marker protein (OMP), a marker for mature OSNs^47^, we found that most of the puncta were localized in the cell bodies of the mature OSNs (Fig. 3 a, b and c).

We took advantage of two available knockout mouse models for stomatin-domain proteins: *i*) a *Triple* KO mouse line, where *Stom*, *Stoml1* and *Stoml3* were knocked out; and *ii*) a *Stoml3* KO mouse line, where only *Stoml3* was knocked out. We confirmed the specificity of our STOM antibody since no staining was observed in *Triple* KO (Fig. 3d–f). Interestingly, by staining the OE of the *Stoml3* KO mouse, the ciliary localization of STOM was not evident anymore, while no changes were observed in the localization of the puncta in OSNs’ cell bodies (Fig. 3g–i). The specific absence of STOM in the *Stoml3* KO mouse did not seem to depend on an alteration of the ciliary layer since acetylated tubulin staining was normal in the different mouse models we analyzed (Suppl. Figure 2). In some OSNs, STOM remained in the apical region of the neuron, fully concentrated in the dendrite and knob, indicating that the absence of STOML3 does not allow STOM to properly localize in the cilia.

**Figure 4:**
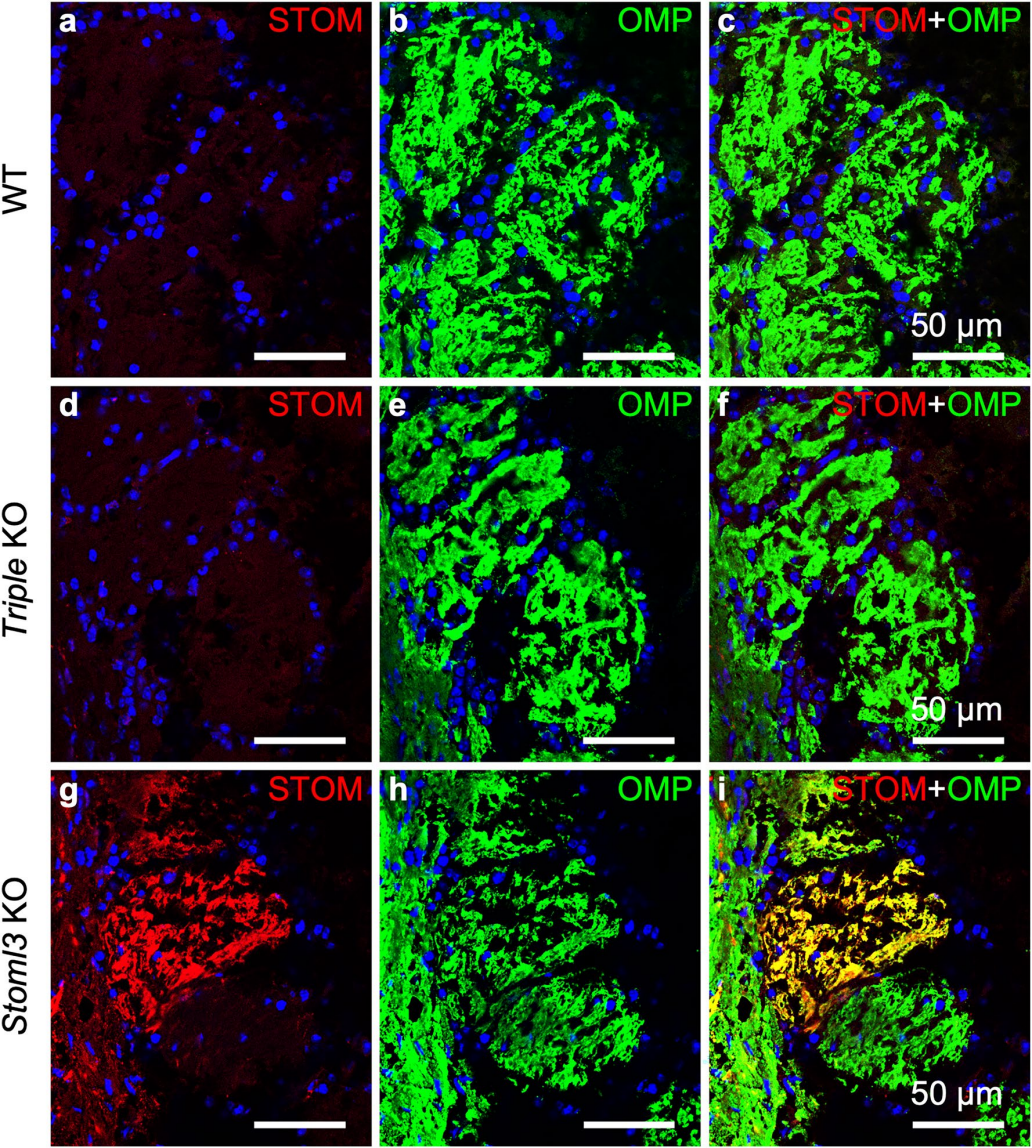
STOM expression in the olfactory bulb. Confocal micrographs of coronal sections of the OB of WT (**a**–**c**), *triple* KO (**d**–**f**), and *Stoml3* KO (**g**–**i**) mice, double-stained with anti-STOM (red) and anti-OMP (in green) antibodies. In WT mice, STOM is not detected in the OB. *Triple* KO (**d**–**f**) mice do not display any staining with anti-STOM antibodies. In contrast, in *Stoml3* KO mice, STOM strongly mis-localizes in the glomeruli (**g**–**i**). Nuclei were stained with DAPI (blue).

OSNs project their axons to the OB in the brain just above the cribriform plate where they synapse in neuropil-like structures called glomeruli. Before reaching the OB, axons coalesce in bundles under the basal lamina of the OE. STOM was not expressed either in the axon bundles or in the glomeruli both in WT and *Triple* KO (Fig. 4a–f). Again, the most surprising finding was that, in the *Stoml*3 KO mouse, STOM re-localized in the most distal part of the axons (Fig. 4g–i). Thus, STOM was found in the OB glomeruli of the *Stoml*3 KO mouse model. Its expression appears non-uniform as we observed a patchy pattern, with some glomeruli showing higher STOM levels than others, also compared to OMP expression (Fig. 4g–i). This staining pattern is reminiscent of that of some guidance molecules expressed in the OB, such as Neuropilin1 or Kirrel2, whose patchy pattern in the OB seems to be dependent respectively from basal and odorant induced activity^48^.

### STOML1

**Figure 5:**
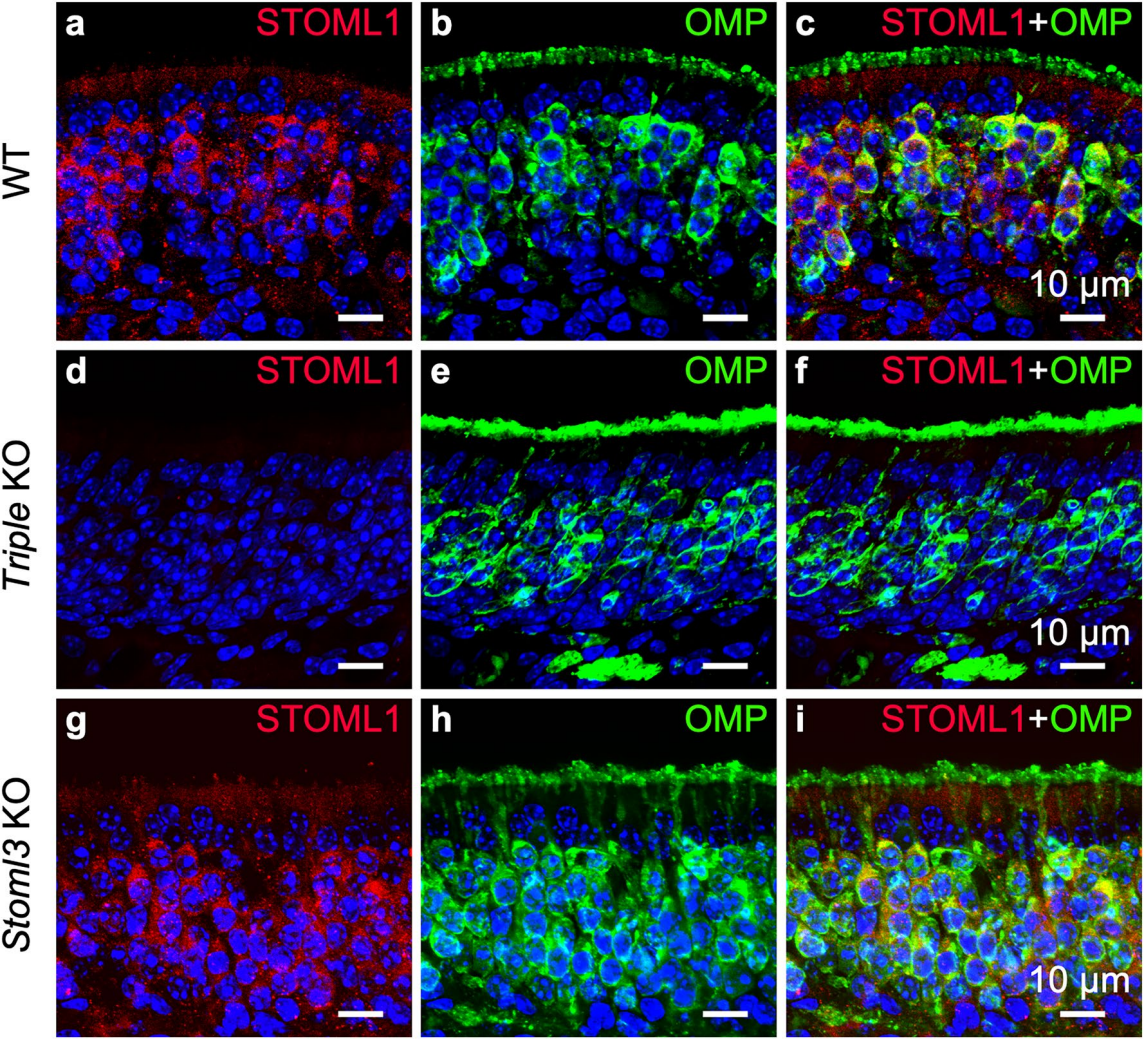
STOML1 is expressed in the cell body of olfactory sensory neurons. Confocal micrographs at high magnification of coronal sections of the olfactory epithelium of WT (**a**–**c**), *triple* KO (**d**–**f**), and *Stoml3* KO (**g**–**i**) mice, double-stained with anti-STOML1 (red) and anti-OMP (green) antibodies. In WT mice, STOML1 localizes in the cell body of the OSNs (**a**–**c**) and same pattern is observed in *Stoml3* KO (**g**–**i**). STOML1 is not detected in *triple* KO mice (**d**–**f** Nuclei were stained with DAPI (blue).

We found that STOML1 is expressed at low levels in the OE (Fig. 2b, f) with higher signals present in structures not directly linked to the peripheral olfactory systems, like the cartilaginous tissue of the septum. Co-staining with OMP showed that the faint STOML1 signal localized mainly in the cell body of mature OSNs and it was absent from the ciliary layer (Fig. 5a–c). Similar to the WT, in the *Stoml3* KO, STOML1 localized in the OSNs’ soma (Fig. 5g–i) while the stain was absent in the *Triple KO* (Fig. 5d–f). No staining was observed in the OB in any of the mouse models used (data not shown).

### STOML2

**Figure 6:**
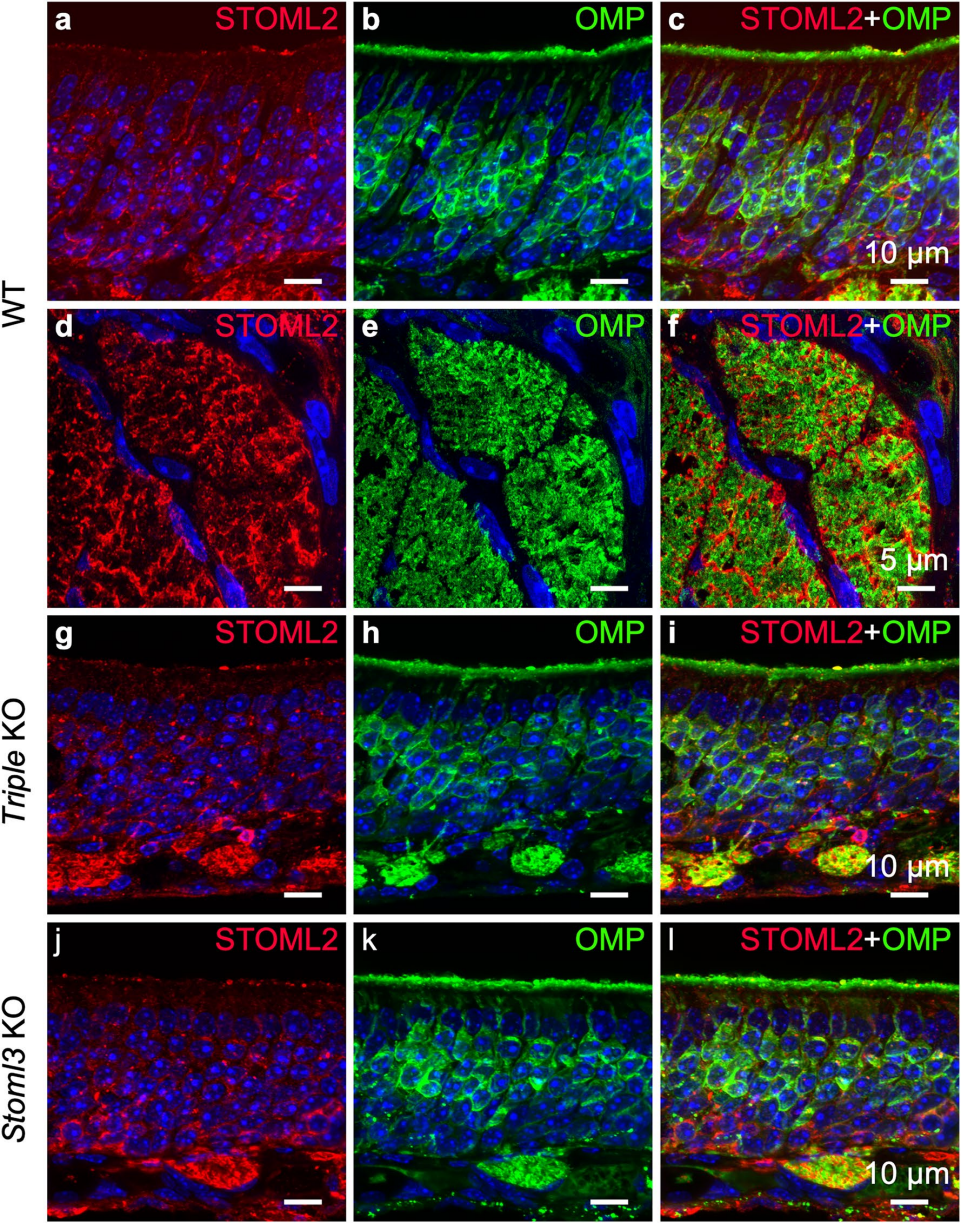
STOML2 is expressed in olfactory sensory neurons, basal stem cells and ensheathing cells. Confocal micrographs at high magnification of coronal sections of the OE of WT (**a**–**c**), *triple* KO (**g**–**i**) and *Stoml3* KO (**j**–**l**) mice, double-stained with anti-STOML2 (red) and anti-OMP (green) antibodies. In all the three mice models, STOML2 has a punctate pattern spread throughout the OE. STOML2 is detected in the soma of the OSNs, although it is almost absent in the ciliary layer. STOML2 signal also is observed in the most basal region of the OE where locate the basal stem cells. (**d**–**f**) Higher magnification of OSN axon bundles shows the localization of STOML2 in ensheathing cells. Nuclei were stained with DAPI (blue).

An interesting aspect that emerged in our characterization of stomatin-domain protein expression in the OE is that each member analyzed had a peculiar expression pattern (Fig. 2). STOML2 further confirmed this aspect. Indeed, its expression was scattered throughout the OE but was almost absent from the ciliary layer (Fig. 6 a, g, j). STOML2 was expressed in the OMP-positive soma in small dots that seemed different from those of STOM, STOML1 and STOML3 (see next paragraph), perhaps indicating that STOML2 had a different intracellular localization (Fig. 6). We also found staining in the most basal region of the OE, where basal stem cells are located. In this region, STOML2 localized around the nuclei occupying all intracellular space and showing a more uniform staining pattern.

STOML2 localization did not seem to be affected by the lack of either *Stom*, *Stoml1* and *Stoml3* together in the *Triple* KO (Fig. 6g–i), or by *Stoml3* alone (Fig. 6j–l).

**Figure 7:**
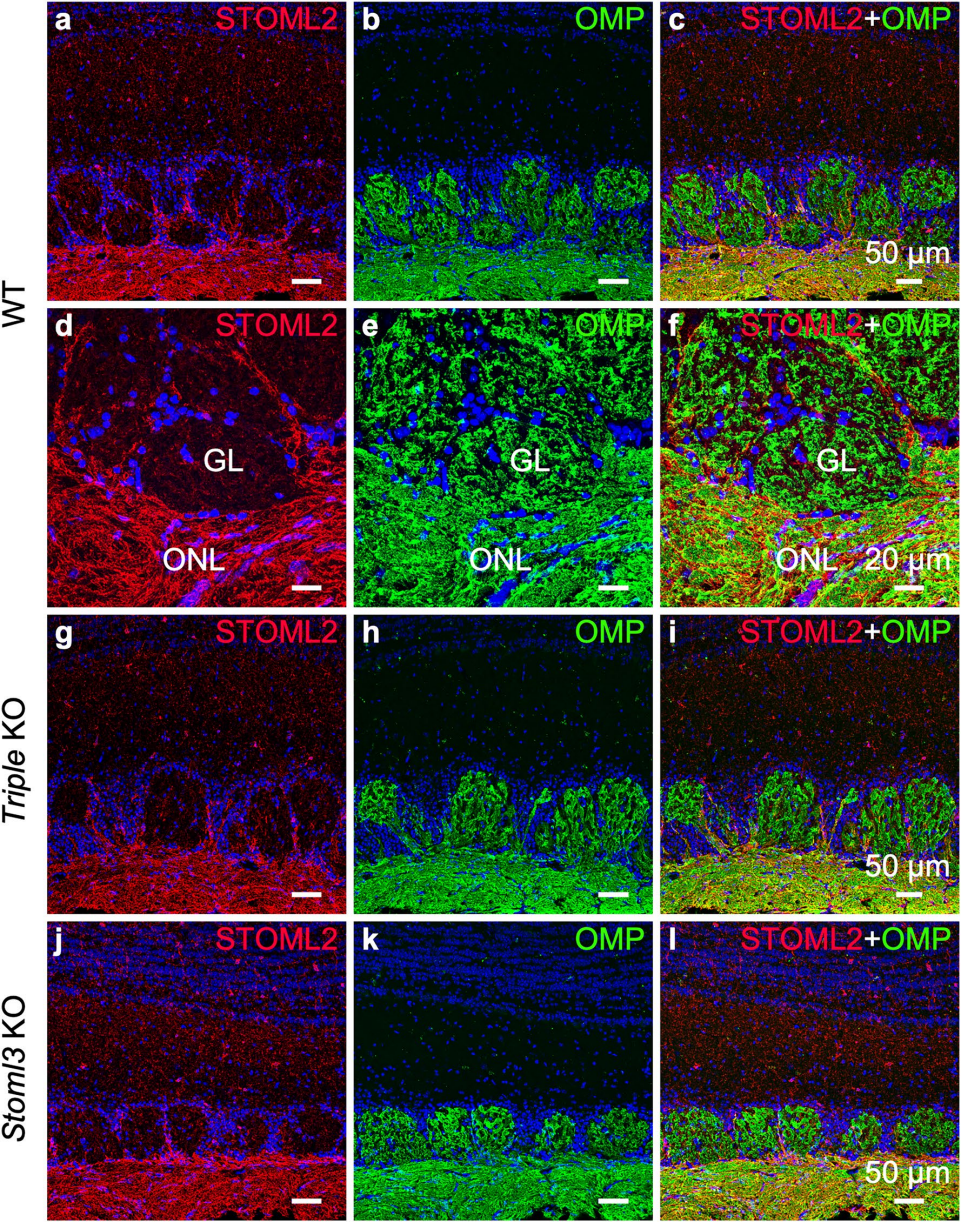
STOML2 is expressed in ensheathing cells but not in olfactory sensory neuron axons. Confocal micrographs of coronal sections of the OB of WT (**a**–**c**), *Triple* KO (**g-i**), and *Stoml3* KO (**j-l**) mice double-stained with anti-STOML2 (red) and anti-OMP (green) antibodies. In all three mice models, STOML2 has a uniform and strong expression in the external nerve layer of the OB while is weakly present in more internal layers including the glomeruli. (**d-f**) Higher magnification of OSN axon bundles shows the localization of STOML2 in ensheathing cells. Nuclei were stained with DAPI (blue).

Interestingly, we observed staining also around the OSN axon bundles, however STOML2 did not colocalized with OMP indicating that it is expressed in the ensheathing cells (Fig. 6d–f). Similarly, in the olfactory bulb STOML2 localized in the olfactory nerve layers without colocalized with OMP suggesting that it also expressed in the population of ensheathing cells enveloping the OSN axons in the bulb (Fig. 7a–f). The lack of either *Stom*, *Stoml1* and *Stoml3* together in the *Triple* KO (Fig. 7g–i), or by *Stoml3* alone (Fig. 7j–l) did not alter the localization of STOML2 in the bulb.

### STOML3

**Figure 8:**
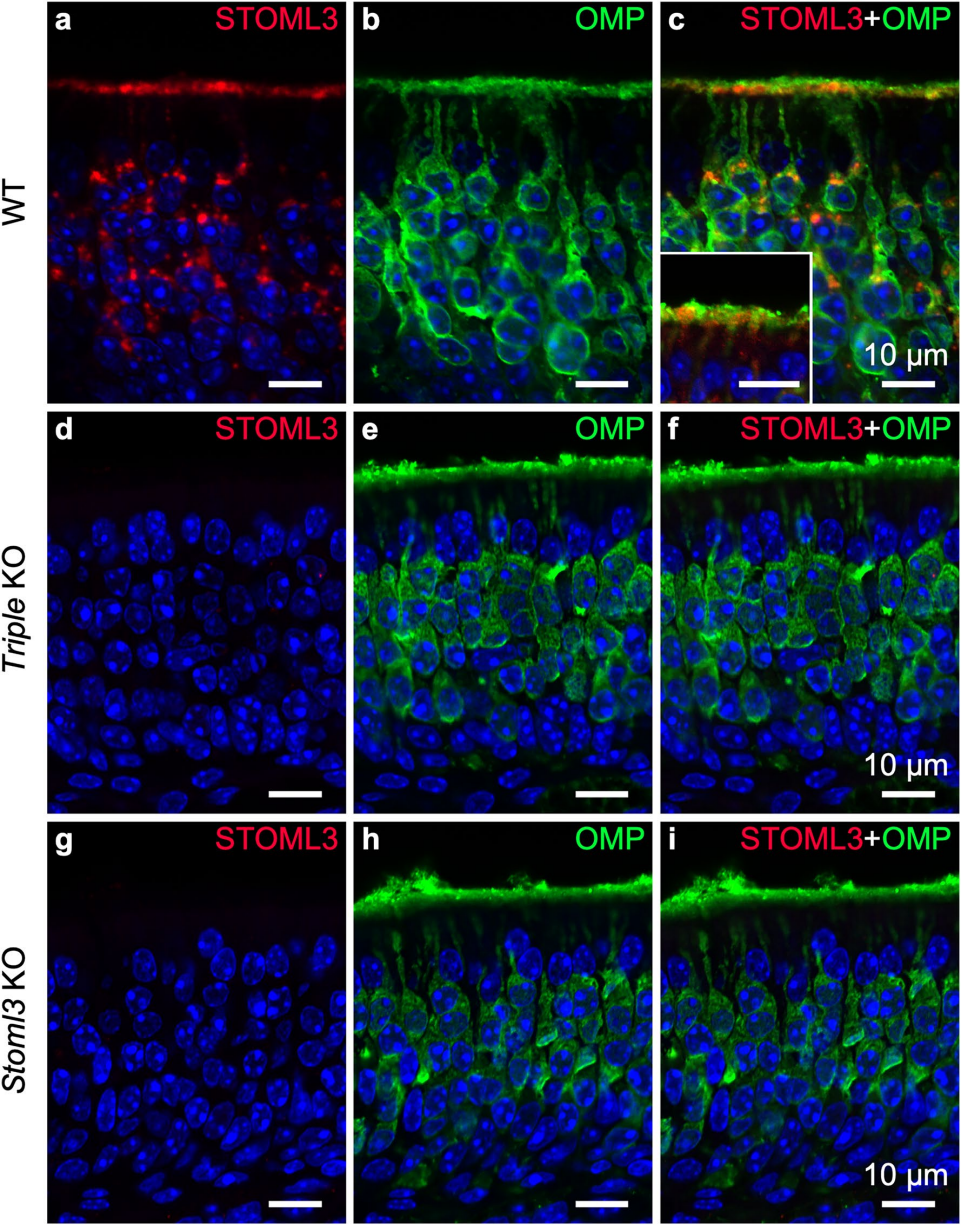
STOML3 expression in the olfactory epithelium. Single optical plane confocal micrographs of coronal sections of the OE of WT (**a**–**c**), *Triple* KO (**d–f**), and *Stoml3* KO (**g-i**) mice, double-stained with anti-STOML3 (red) and anti-OMP (green) antibodies. In WT mice, STOML3 predominantly localizes in a continuous thin layer located along the basal portion of the cilia and knobs of OSNs. The inset in **c** shows the localization of STOML3 in the knob/basal part of the cilia. Resembling STOM expression, STOML3 also is detected in several puncta in the cell body of most OSNs. STOML3 signal is not visible in triple KO and *Stoml3* KO mice, confirming the specificity of the signal observed in WT. Nuclei were stained with DAPI (blue). Inset scale bar 10 μm.

We have recently shown that STOML3 is expressed in the OE and it is enriched in the knob and the proximal part of the OSN cilia (Suppl. Figure 2 and^7^). The STOML3 positive roundish apical cellular structures are suggestive of knobs of mature OSNs (Fig. 8a–c and inset). This staining pattern vanished in the *Triple* KO and in *Stoml*3 KO mice confirming the specificity of the immunostaining (Fig. 8). STOML3 also localized in OSNs’ cell bodies with a rather intracellular punctate pattern.

STOML3 was not expressed in the axon bundles, and we could not detect any signal in the glomerular layer. We found a staining in other regions of the OB, but this signal was also present in the KO models (data not shown). Because the used antibodies are pretty specific inside the stomatin-domain family, the likely explanation for the staining in the OB of the *Stoml*3-KO and *Triple* KO, is a cross-reactivity of the STOML3 antibody with a non-stomatin-domain protein.

### STOM and STOML3 vesicular localization

**Figure 9:**
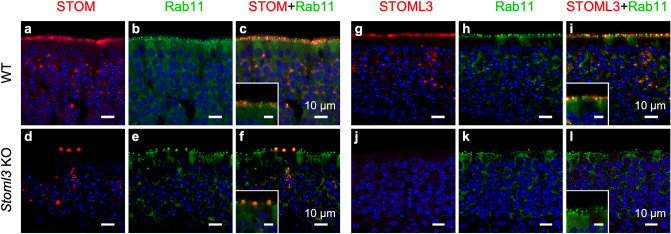
STOM and STOML3 co-localize with the endosomal marker Rab11. Single optical plane confocal micrographs of coronal sections of the OE of WT and *Stoml3* KO mice stained with antibodies against STOM (**a** and **d**, red) or STOML3 (**g** and **j**, red). The same slices were double-stained with anti-Rab11 (**b**, **e**, **h**, and **k**, green). As described before, STOM and STOML3 mainly localize in the cilia (see insets) and in puncta in the cell body of OSNs. Rab11 is scattered throughout the OE, although it looks particularly enriched apically in the knobs of OSNs **(b**, **e**, **h**, and **k)** where both STOM (**a**, **d**, **c** and **f**) and STOML3 (**g**, **j**, **i** and **l**) localize. Nuclei were stained with DAPI (blue). Insets scale bar 10 μm.

The puncta observed for STOM and STOML3 immunostaining in the central portion of the OE were reminiscent of intracellular vesicles. Indeed, the two proteins did not seem to be expressed uniformly on the cell membrane of OSNs' cell bodies but rather intracellularly (Figs. 3, 8, 9). To better understand the localization of STOM and STOML3 we used the endosomal marker Rab11, that belong to a class of Ras GTPases that regulate vesicle-based intracellular transport ^15,49^. We found that Rab11 co-localized with STOM and STOML3 (Fig. 9a–l). The expression of Rab11 was quite scattered but interestingly Rab11 positive vesicles had a higher density in the apical layer, where dendritic knob and cilia of OSN are located, than in the central/basal portion, where cell bodies of OSNs are located.

In the central portion of the OE, Rab11 vesicles seemed smaller than the pools of STOM/STOML3, being the former fully contained in the latter. As these vesicles are highly mobile^15^, the intense staining and the only partial overlap between STOM/STOML3 and Rab11 in the apical layer may indicate a continuous turnover between the membrane and the tiny intracellular space.

In the apical region of the OE, we observed a more intense staining for Rab11 (a higher density of vesicles) that was partially overlapping with STOM or STOML3. Interestingly, the knob region of some OSNs contained only Rab11 positive vesicles, while knobs of other OSNs contained both Rab11 and STOM (Fig. 9c, f and e), or Rab11 and STOML3 (Fig. 9i) positive vesicles.

As mentioned above, STOML3 is necessary for STOM to correctly target to the knob/proximal cilia region of OSNs (Fig. 3g–i). In *Stoml3* KO, Rab-11 positive vesicles are still present throughout the OE as in WT mice, and especially those in the knob/cilia are mostly devoid of STOM (and of course of STOML3) proteins, indicating that STOML3 protein is necessary for a correct vesicular transport of STOM proteins. In the *Stoml3* KO, few knobs contained STOM that was only partially overlapping with Rab11 indicating that, although STOML3 is necessary for STOM to normally localize in the knob and proximal cilia membrane, not all of it remains trapped in the vesicles and can reach the membrane.

## Discussion

Here we sought to investigate the expression pattern of stomatin-domain proteins in the mouse OE and OB. We found that four out of five members are expressed in different structures and cell types. By a quick survey of the transcriptomic data available in repositories we could find that *Stom* and *Stoml3* are among the highest expressed transcripts in the mouse OE, while *Stoml1* and *Stoml2* expression levels seem lower^45,46,50^. The high expression of *Stom* and *Stoml3* transcripts in the OE has been confirmed by qRT-PCR by Wetzel et al.^18^, who also pointed out that, among several tested tissues, the OE has the highest expression levels for these two transcripts. In addition, several proteomics studies have been focused on the detection of the proteins in the cilia of the OSNs by using biochemically isolated cilia extracts from the OE of mice and rats. All studies agreed on the presence of STOM and STOML3 in the cilia extracts^41,42,44,51^. Moreover, Tadenev et al.^11^ using an eGFP reporting mice showed that STOML3 is localized in the knob and in the proximal portion of the cilia (see Fig. 4 from^11^). Here, we showed for the first time where four members of the stomatin-domain proteins localize in the OE and OB.

A first interesting finding is that STOM localization in OSNs depends on STOML3 expression. Indeed, when knocking out *Stoml3*, STOM does not localize on the knob/cilia of the OSNs but it rather remains in the cell body. In a few cases, we even observed STOM trapped in the knobs. By using Rab11, a member of the Rab GTPase family of proteins that play a major role in the transport of important signaling proteins to the plasma membrane^52,53^, we observed that both STOM and STOML3 were present in vesicles. Förster resonance energy transfer (FRET) experiments showed that STOM and STOML3 physically interact in intracellular vesicles in DRG neurons^15^. Moreover, in DRG neurons STOML3-containing vesicles express other transduction molecules, such as ASICs channels, suggesting that this type of vesicles is involved in assembly and trafficking to plasma membrane of “transducosome”^15^. We do not know whether Rab11 positive vesicles contained olfactory transduction proteins, even though it seems that at least odorant receptors are transported in OSNs along the dendrite to the cilia via Rab11 negative vesicles^53^. When STOML3 was missing, we could find Rab11 positive vesicles densely packed in the knobs, indicating that they are important for the incorporation of yet to be known interacting ciliary proteins. As previously proposed for DRG neurons^15^, it is tempting to speculate that Rab11 positive vesicles might represent nascent “transducosome” ready to shuttle ciliary proteins at their final destination also allowing for the highly compartmentalized expression of the stomatin-domain proteins. However, Rab11 is well known to be involved in endocyting vesicular trafficking to the plasma membrane^52,53^.

Even more striking was the finding that when STOML3 was absent, STOM localized in the OSNs’ axons in OB glomeruli (where normally it is not expressed) thus showing that STOML3 is needed for a proper STOM localization also in the OB. Moreover, STOM distribution in *Stoml3* KO OB is not uniform, but it seems that different glomeruli have different STOM expression level.

STOM accumulation in the OSN’ axons in the *Stoml3* KO mice could be simply an epiphenomenon due to the inability of the STOM protein to properly localize. Another possible explanation of the patchy staining pattern is that STOM localization in the OB of the *Stoml3* KO may correlate with guidance molecules like Kirrel2/3 or Neuropilin1 or others^54^. Indeed, several guidance molecules have peculiar staining pattern. For example, it has been shown that differing firing patterns induced the expression of different axon-sorting molecules regulating axonal segregation in mice^55^. Phasic, repetitive short bursts of neural activity induced Kirrel2 expression, whereas bursting activity induced the expression of Sema7A and PCDH10 molecules that are involved in axon sorting for OR specific glomerular formation^55^.

Recently, we have shown that STOML3 may modulate action potential firing is OSNs in response to signal transduction activation. In *Stoml3* KO OSNs, action potential firing was reduced both in spontaneous activity and in odorant induced responses. Now, in light of the results presented here, we could complete the picture by introducing STOM that may cooperate with STOML3 in modulating OSNs’ firing output^7^ and ultimately in glomerular formation by altering the levels of the expression of the afore-mentioned guidance molecules. Although we could not observe any evident change in glomerular targeting and formations in the OB, the role of STOM-STOML3 in the olfactory system is certainly worth further investigation.

At a first glance, STOM and STOML3 seemed to have a similar expression pattern in OSNs, especially in the knob/cilia region, while STOML1 is more abundant in the cartilaginous tissue of the septum than in the OE, and STOML2 is expressed not only in the OE but also in the axon bundles beneath the basal lamina. STOML1 expression seemed the lowest among the four proteins in the OE. It showed a faint punctate expression pattern in OSN cell bodies, and the antibody revealed a perinuclear staining and a punctate pattern throughout the cytoplasm compatible with vesicles and other membranous organelles^54^.

STOML2 is probably the least described member of the stomatin-domain protein family. STOML2 has been found in many cell types in the inner mitochondrial membrane^30,33^. In neurons, it localizes in plasma membrane (Kozlenkov, Lapatsina and Lewin, unpublished). In pathological conditions, STOML2 promotes tumor progression and cell proliferation^35–37^. We found that STOML2 is expressed in the basal cells of the OE, but we could not determine whether it is expressed in mitochondria or in the plasma membrane. Interestingly, it is the only member out of four to be expressed both in mature OSNs and in basal cells, that constitute the stem cell niche of the OE ready to differentiate in various cell types, including OSNs^56–58^. In addition, STOML2 abundantly localizes in the ensheathing cells both beneath the olfactory epithelium and in outer nerve layer of the OB. The ensheathing cells are glia cell type that envelopes the OSN axons and could play an important role in sustain and guide the axons of the regenerating neurons to the OB^59,60^. Many studies show the potential use of the ensheathing cells to support neuronal repair after injury^61^.

In summary, by addressing the expression and localization of stomatin-domain proteins in OE and OB we showed that: i) STOM needs STOML3 to correctly target in the knob/cilia of OSNs; ii) In OSNs, STOM and STOML3 are also present in Rab11 positive vesicles that represent a cargo system that may form a nascent transducisome that brings transduction proteins to the neurons’ apical region; iii) STOML1 is expressed in the cartilage of the septum and at a lower level in OSNs’ cell body; and iv) STOML2 has a very peculiar localization pattern, being expressed in basal cells as well as in OSNs’ cell bodies and in the ensheathing cells.

## Materials and methods

### Animals

Mice aged 2–6 months of both sexes were handled in accordance with the Italian Guidelines for the Use of Laboratory Animals and the European Union guidelines on animal research according to a protocol approved by the ethics committee of Scuola Internazionale Superiore di Studi Avanzati (SISSA). Experiments were performed on tissues from C57BL/6 wild-type, *Stoml3* KO (Wetzel et al., 2007) and triple KO mice for *Stom*, *Stoml1* and *Stoml3*^18,26,27^.

### RT-PCR

mRNA was extracted from 10 to 20 mg of the olfactory epithelium of 2 wild type (WT) mice using magnetic beads mRNA isolation kit (S1550S, New England Biolabs). After extraction, mRNA was further incubated with DNAase I (M0303S, New England Biolabs) to remove any residual genomic DNA. cDNA was synthetized using the Protoscript II First-Strand DNA Synthesis Kit from 100 ng of mRNA (E6560S, New England Biolabs). PCR was performed in thermocycler (ThermaCycler2720, LifeTechnologies) using Taq DNA polymerase and Thermopol buffer (M0267S, New England Biolabs), 0.2 mM for each dNTPs (N0447S, New England Biolabs) and 200 pmol forward/reverse target-specific primers. Cycling parameters were: an initial denaturation step (95 °C, 2 min) followed by 36 cycles, each of these cycles included a denaturation step (95 °C, 30 s), a primer annealing step (62–64 °C, 30 s), and an extension step (72 °C, 60 s). Reaction was completed by a final extension step at 72 °C for 5 min. Contamination from genomic DNA was tested by using a sample of the mRNA without retrotranscription step. cDNA from testis (Clontech Laboratories) was used to set a positive control for PCR amplification to confirm that the primers of *Podocin* are properly working. The following primers were used: *Gapdh* fwd 5'-TGCTGAGTATGTCGTGGAGTCT-3' rev 5'-TGCTGTAGCCGTATTCATTGTC-3' (Tm = 62 °C; 691 bp; GenBank. accession no. NM_008084.3); *Olfr73* fwd 5'-GCTGGTATTGGGATCCTATGCTT-3' rev 5'- CGTCCACTTGCTGACTTCATCTT-3' (Tm = 62 °C; 272 bp; GenBank. accession no. NM_054090.1); *G*_*αOLF*_ fwd 5'-CTGCACGTCAATGGCTTCAA-3' rev 5'-TCACGGCAATCGTTGAACAC-3' (Tm = 64 °C; 910 bp; GenBank. accession no. NM_177137.5); *Stom* fwd 5'-AAGACAGAACTGGGAGCTTGTG-3' rev 5'-TGATAACCATGGACGCTTCTTTC-3' (Tm = 64 °C; 654 bp; GenBank. accession no. X91043.1); *Stoml1* fwd 5'-GGATGATTGTGTTTCGACTGG-3' rev 5'-TTATCATCTCCAAGGTGTCTGG-3' (Tm = 64 °C; 548 bp; GenBank. accession no. XM_006511402.4); *Stoml2* 5'-TACAAGGCAAGTTACGGTGTGG-3' rev 5'-GAGAATGCGCTGACATACTGCT-3' (Tm = 64 °C; 523 bp; GenBank. accession no. AK002793); *Stoml3* 5'-CCCAATCTCAGTATGGATGTGC-3' rev 5'-AGGCTTTAGCAGTGACCTTCTTAT-3' (Tm = 64 °C; 745 bp; GenBank. accession no. NM_153156.2); *Podocin* 5'-CATCAAGCCCTCTGGATTAGG-3' rev 5'-AGCGACTGAAGAGTGTGCAAGT-3' (Tm = 64 °C; 713 bp; GenBank. accession no. AJ302048).

### Immunohistochemistry

The anterior part of the head containing the nasal cavity and the olfactory bulb was fixed in 4% paraformaldehyde in PBS pH 7.4 for 4 h at 4 °C. After fixing, heads of adult mice were decalcified in 0.5 M EDTA pH 8 for 2–4 days. For cryoprotection tissue was equilibrated overnight in 30% (w/v) sucrose in PBS at 4 °C. Then, the tissues were embedded in cryostat embedding medium (BioOptica) and immediately frozen at − 80 °C. 14–16 μm coronal sections were cut on a cryostat and mounted on Superfrost Plus Adhesion Microscope Slides (ThermoFisher Scientific). Sections were air-dried for 3 h and used the same day or stored at -20 °C for further use. To remove the cryostat embedding medium from tissue, the slices were incubated for 15 min with PBS. Tissue was treated for 15 min with 0.5% (w/v) sodium dodecyl sulfate (SDS) in PBS for antigen retrieval, then washed and incubated in blocking solution (2% normal donkey serum, 0.2% Triton X-100 in PBS) for 90 min and finally overnight at 4 °C in primary antibodies diluted in blocking solution. As primary antibodies we used (company; catalog number; dilution): polyclonal rabbit anti-STOM (Sigma; HPA011419; 1:200); polyclonal rabbit anti-STOML1 (Sigma; HPA042353; 1:200); polyclonal rabbit anti-STOML2 (Abcam; ab191883; 1:200); polyclonal rabbit anti-STOML3 (Proteintech; 13,316–1-AP; 1:200); polyclonal goat anti-OMP (Wako; 544–10,001; 1:1000); polyclonal goat anti-Rab11 (AB3035-200, Origene; 1:200). After removal of the excess of primary antibodies with PBS washes, sections were incubated with Alexa Fluor-conjugated secondary antibodies (1:500 dilution) in TPBS (Tween 20 0.2% in PBS) for 2 h at room temperature, washed and mounted with Vectashield (Vector Laboratories) or Fluoromount-G (ThermoFisher). The following secondary antibodies were used: donkey anti-rabbit Alexa Fluor Plus 594 (Life Technologies; A32754; 1:500) and donkey anti-goat Alexa Fluor 488 (Life Technologies; A11055; 1:500). DAPI (5 μg/ml) was added in solution containing secondary antibody to stain the nuclei. To reveal antibodies anti STOML1 and STOML3 we applied the tyramide signal amplification method using the Tyramide SuperBoost™ Kit (B40925, ThemoFisher;^62^). Immunofluorescence was visualized with a confocal laser scanning microscope (A1R, Nikon). Images were acquired using NIS-Elements Nikon software at 1024 × 1024 pixels resolution and analyzed with ImageJ software (National Institute of Health, USA). For images in Figs. 2, 3, 4, 5, 6 and 7, the maximum projections of 3–6 optical slices were displayed. Control experiments, excluding primary antibodies, were performed for each immunolocalization that was not possible to test in knockout mice and gave no signal.

### Cell culture, transfection and immunocytochemistry

HEK-293 (Sigma) were grown in medium composed of DMEM (Gibco, Italy) supplemented with 10% FBS (Sigma, Italy), 100 IU/ml penicillin and 100 μg/ml streptomycin (Sigma, Italy) at 37 °C in a humidified atmosphere of 5% CO2. Cells were transfected with X-tremeGENE 9 DNA Transfection Reagent (Roche) following the protocol recommended by the supplier. mCherry or EGFP-fusion stomatin-domain protein constructs were used to transfect the cells. In particular, we used STOM-mCherry, STOML1-mCherry, STOML2-EGFP and STOML3-mCherry^15^. After 24–48 h from transfection the cells were plated on circular coverslips coated with poly-L-lysine to improve the adhesion and used for immunocytochemistry experiments.

Transfected cells were fixed with 4% paraformaldehyde in PBS pH 7.4 for 20 min at room temperature. Cells were incubated in quenching solution (0.1 M of glycine) for 15 min at room temperature, then permeabilized 0.01% Triton X-100 in PBS for 15 min and washed. After incubation with blocking solution (2% FBS, 0.2% Triton X100 in PBS) for 15 min, cells were incubated in primary antibodies diluted in blocking solution for 4 h at 4 °C. As primary antibodies, we used the same as in experiments of immunohistochemistry. After removal of unconjugated primary antibodies with PBS washes, cells were incubated with Alexa Fluor-conjugated secondary antibodies (1:500 dilution) in TPBS (Tween 20 0.2% in PBS) for 45 min at room temperature, washed and mounted with Vectashield (Vector Laboratories) or Fluoromount-G (ThemoFisher). DAPI (5 μg/ml) was added in solution containing secondary antibody to stain the nuclei. The following secondary antibodies were used: donkey anti-rabbit Alexa Fluor Plus 594 (Life Technologies; A32754; 1:500) and donkey anti-rabbit Alexa Fluor 488 (Life Technologies; A21206; 1:500). Image acquisition was made similar as in the experiments of immunohistochemistry.

### Ethics approval

The study is reported in accordance with ARRIVE guideline.

## Supplementary Information

Supplementary Information.

## Data Availability

The data that support the findings of this study are available from the corresponding author upon reasonable request.

## References

[1] Boccaccio A, Lagostena L, Hagen V, Menini A (2006). Fast adaptation in mouse olfactory sensory neurons does not require the activity of phosphodiesterase. J. Gen. Physiol..

[2] Dibattista M, Pifferi S, Boccaccio A, Menini A, Reisert J (2017). The long tale of the calcium activated Cl- channels in olfactory transduction. Channels Austin Tex.

[3] Kleene SJ (2008). The electrochemical basis of odor transduction in vertebrate olfactory cilia. Chem. Senses.

[4] Menini A, Lagostena L, Boccaccio A (2004). Olfaction: From odorant molecules to the olfactory cortex. News Physiol. Sci. Int. J. Physiol. Prod. Jointly Int Union Physiol. Sci. Am. Physiol. Soc..

[5] Pifferi S, Cenedese V, Menini A (2012). Anoctamin 2/TMEM16B: A calcium-activated chloride channel in olfactory transduction. Exp. Physiol..

[6] Schild D, Restrepo D (1998). Transduction mechanisms in vertebrate olfactory receptor cells. Physiol. Rev..

[7] Agostinelli E (2021). A Role for STOML3 in olfactory sensory transduction. eNeuro.

[8] Goldstein BJ, Kulaga HM, Reed RR (2003). Cloning and characterization of SLP3: A novel member of the stomatin family expressed by olfactory receptor neurons. J. Assoc. Res. Otolaryngol. JARO.

[9] Kobayakawa K (2002). Stomatin-related olfactory protein, SRO, specifically expressed in the murine olfactory sensory neurons. J. Neurosci. Off. J. Soc. Neurosci..

[10] Kulaga HM (2004). Loss of BBS proteins causes anosmia in humans and defects in olfactory cilia structure and function in the mouse. Nat. Genet..

[11] Tadenev ALD (2011). Loss of Bardet-Biedl syndrome protein-8 (BBS8) perturbs olfactory function, protein localization, and axon targeting. Proc. Natl. Acad. Sci. U. S. A..

[12] Lapatsina L, Brand J, Poole K, Daumke O, Lewin GR (2012). Stomatin-domain proteins. Eur. J. Cell Biol..

[13] Brand J (2012). A stomatin dimer modulates the activity of acid-sensing ion channels. EMBO J..

[14] Tavernarakis N, Driscoll M, Kyrpides NC (1999). The SPFH domain: implicated in regulating targeted protein turnover in stomatins and other membrane-associated proteins. Trends Biochem. Sci..

[15] Lapatsina L (2012). Regulation of ASIC channels by a stomatin/STOML3 complex located in a mobile vesicle pool in sensory neurons. Open Biol..

[16] Poole K, Herget R, Lapatsina L, Ngo H-D, Lewin GR (2014). Tuning Piezo ion channels to detect molecular-scale movements relevant for fine touch. Nat. Commun..

[17] Wetzel C (2017). Small-molecule inhibition of STOML3 oligomerization reverses pathological mechanical hypersensitivity. Nat. Neurosci..

[18] Wetzel C (2007). A stomatin-domain protein essential for touch sensation in the mouse. Nature.

[19] Stewart GW (1992). Isolation of cDNA coding for an ubiquitous membrane protein deficient in high Na+, low K+ stomatocytic erythrocytes. Blood.

[20] Fricke B (2003). The ‘stomatin’ gene and protein in overhydrated hereditary stomatocytosis. Blood.

[21] Bawazir WM (2012). An infant with pseudohyperkalemia, hemolysis, and seizures: Cation-leaky GLUT1-deficiency syndrome due to a SLC2A1 mutation. J. Clin. Endocrinol. Metab..

[22] Flatt JF, Bruce LJ (2018). The molecular basis for altered cation permeability in hereditary stomatocytic human red blood cells. Front. Physiol..

[23] Albuisson J (2013). Dehydrated hereditary stomatocytosis linked to gain-of-function mutations in mechanically activated PIEZO1 ion channels. Nat. Commun..

[24] Zarychanski R (2012). Mutations in the mechanotransduction protein PIEZO1 are associated with hereditary xerocytosis. Blood.

[25] Mannsfeldt AG, Carroll P, Stucky CL, Lewin GR (1999). Stomatin, a MEC-2 like protein, is expressed by mammalian sensory neurons. Mol. Cell. Neurosci..

[26] Moshourab RA, Wetzel C, Martinez-Salgado C, Lewin GR (2013). Stomatin-domain protein interactions with acid-sensing ion channels modulate nociceptor mechanosensitivity. J. Physiol..

[27] Kozlenkov A, Lapatsina L, Lewin GR, Smith ESJ (2014). Subunit-specific inhibition of acid sensing ion channels by stomatin-like protein 1. J. Physiol..

[28] Wang Y, Morrow JS (2000). Identification and characterization of human SLP-2, a novel homologue of stomatin (band 7.2b) present in erythrocytes and other tissues. J. Biol. Chem..

[29] Kirchhof MG (2008). Modulation of T cell activation by stomatin-like protein 2. J. Immunol. Baltim. Md.

[30] Hájek P, Chomyn A, Attardi G (2007). Identification of a novel mitochondrial complex containing mitofusin 2 and stomatin-like protein 2. J. Biol. Chem..

[31] Christie DA (2011). Stomatin-like protein 2 binds cardiolipin and regulates mitochondrial biogenesis and function. Mol. Cell. Biol..

[32] Mitsopoulos P (2015). Stomatin-like protein 2 is required for in vivo mitochondrial respiratory chain supercomplex formation and optimal cell function. Mol. Cell. Biol..

[33] Ikon N, Ryan RO (2017). Cardiolipin and mitochondrial cristae organization. Biochim. Biophys. Acta Biomembr..

[34] Christie DA, Kirchhof MG, Vardhana S, Dustin ML, Madrenas J (2012). Mitochondrial and plasma membrane pools of stomatin-like protein 2 coalesce at the immunological synapse during T cell activation. PLoS ONE.

[35] Yang X (2018). The diagnostic value of TROP-2, SLP-2 and CD56 expression in papillary thyroid carcinoma. Eur. Arch. Oto-Rhino-Laryngol. Off. J. Eur. Fed. Oto-Rhino-Laryngol. Soc. EUFOS Affil. Ger. Soc. Oto-Rhino-Laryngol. - Head Neck Surg..

[36] Zhou C (2019). Enhanced SLP-2 promotes invasion and metastasis by regulating Wnt/β-catenin signal pathway in colorectal cancer and predicts poor prognosis. Pathol. Res. Pract..

[37] Zheng Y (2021). STOML2 potentiates metastasis of hepatocellular carcinoma by promoting PINK1-mediated mitophagy and regulates sensitivity to lenvatinib. J. Hematol. Oncol. J Hematol Oncol.

[38] Roselli S (2002). Podocin localizes in the kidney to the slit diaphragm area. Am. J. Pathol..

[39] Boute N (2000). NPHS2, encoding the glomerular protein podocin, is mutated in autosomal recessive steroid-resistant nephrotic syndrome. Nat. Genet..

[40] Yu T-T (2005). Differentially expressed transcripts from phenotypically identified olfactory sensory neurons. J. Comp. Neurol..

[41] Mayer U (2008). Proteomic analysis of a membrane preparation from rat olfactory sensory cilia. Chem. Senses.

[42] Mayer U (2009). The proteome of rat olfactory sensory cilia. Proteomics.

[43] Klimmeck D (2008). Calcium-signaling networks in olfactory receptor neurons. Neuroscience.

[44] Stephan AB (2009). ANO2 is the cilial calcium-activated chloride channel that may mediate olfactory amplification. Proc. Natl. Acad. Sci. U. S. A..

[45] Ibarra-Soria X, Levitin MO, Saraiva LR, Logan DW (2014). The olfactory transcriptomes of mice. PLoS Genet..

[46] Kanageswaran N (2015). Deep sequencing of the murine olfactory receptor neuron transcriptome. PLoS ONE.

[47] Keller A, Margolis FL (1975). Immunological studies of the rat olfactory marker protein. J. Neurochem..

[48] Dibattista M, Reisert J (2016). The odorant receptor-dependent role of olfactory marker protein in olfactory receptor neurons. J. Neurosci. Off. J. Soc. Neurosci..

[49] Grosshans BL, Ortiz D, Novick P (2006). Rabs and their effectors: Achieving specificity in membrane traffic. Proc. Natl. Acad. Sci. U. S. A..

[50] Saraiva LR (2015). Hierarchical deconstruction of mouse olfactory sensory neurons: from whole mucosa to single-cell RNA-seq. Sci. Rep..

[51] Kuhlmann K (2014). The membrane proteome of sensory cilia to the depth of olfactory receptors. Mol. Cell. Proteomics MCP.

[52] Ullrich O, Reinsch S, Urbé S, Zerial M, Parton RG (1996). Rab11 regulates recycling through the pericentriolar recycling endosome. J. Cell Biol..

[53] Schlierf B, Fey GH, Hauber J, Hocke GM, Rosorius O (2000). Rab11b is essential for recycling of transferrin to the plasma membrane. Exp. Cell Res..

[54] Sakano H (2020). Developmental regulation of olfactory circuit formation in mice. Dev. Growth Differ..

[55] Nakashima A (2019). Structured spike series specify gene expression patterns for olfactory circuit formation. Science.

[56] Graziadei PP, Graziadei GA (1979). Neurogenesis and neuron regeneration in the olfactory system of mammals. I. Morphological aspects of differentiation and structural organization of the olfactory sensory neurons. J. Neurocytol..

[57] Huard JM, Schwob JE (1995). Cell cycle of globose basal cells in rat olfactory epithelium. Dev. Dyn. Off. Publ. Am. Assoc. Anat..

[58] Schwob JE (2017). Stem and progenitor cells of the mammalian olfactory epithelium: Taking poietic license. J. Comp. Neurol..

[59] Graziadei PP, Monti Graziadei GA (1980). Neurogenesis and neuron regeneration in the olfactory system of mammals. III. Deafferentation and reinnervation of the olfactory bulb following section of the fila olfactoria in rat. J. Neurocytol..

[60] Rieger A, Deitmer JW, Lohr C (2007). Axon-glia communication evokes calcium signaling in olfactory ensheathing cells of the developing olfactory bulb. Glia.

[61] Ursavas S, Darici H, Karaoz E (2021). Olfactory ensheathing cells: Unique glial cells promising for treatments of spinal cord injury. J. Neurosci. Res..

[62] Hunyady B, Krempels K, Harta G, Mezey E (1996). Immunohistochemical signal amplification by catalyzed reporter deposition and its application in double immunostaining. J. Histochem. Cytochem. Off. J. Histochem. Soc..

