# Supplementary material for: Expression pattern of Stomatin-domain proteins in the peripheral olfactory system
Source: Sci Rep. 2022 Jul 6;12:11447. doi: 10.1038/s41598-022-15572-1 (PMC9259621; doi:10.1038/s41598-022-15572-1)
Supplement: Supplementary file 1 — Supplementary Information. [file 41598_2022_15572_MOESM1_ESM.pdf]

Expression pattern of Stomatin-domain proteins in the peripheral olfactory system

Kevin Y. Gonzalez-Velandia[1], Andres Hernandez-Clavijo[1], Anna Menini[1]*, Michele Dibattista[2*], Simone Pifferi[1,3]*

[1] Neurobiology Group, SISSA, Scuola Internazionale Superiore di Studi Avanzati, 34136 Trieste, Italy.
[2] Department of Basic Medical Sciences, Neuroscience and Sensory Organs, University of Bari A.
Moro, 70124 Bari, Italy
[3] Department of Experimental and Clinical Medicine, Università Politecnica delle Marche, 60126 Ancona, Italy.

*Corresponding Authors

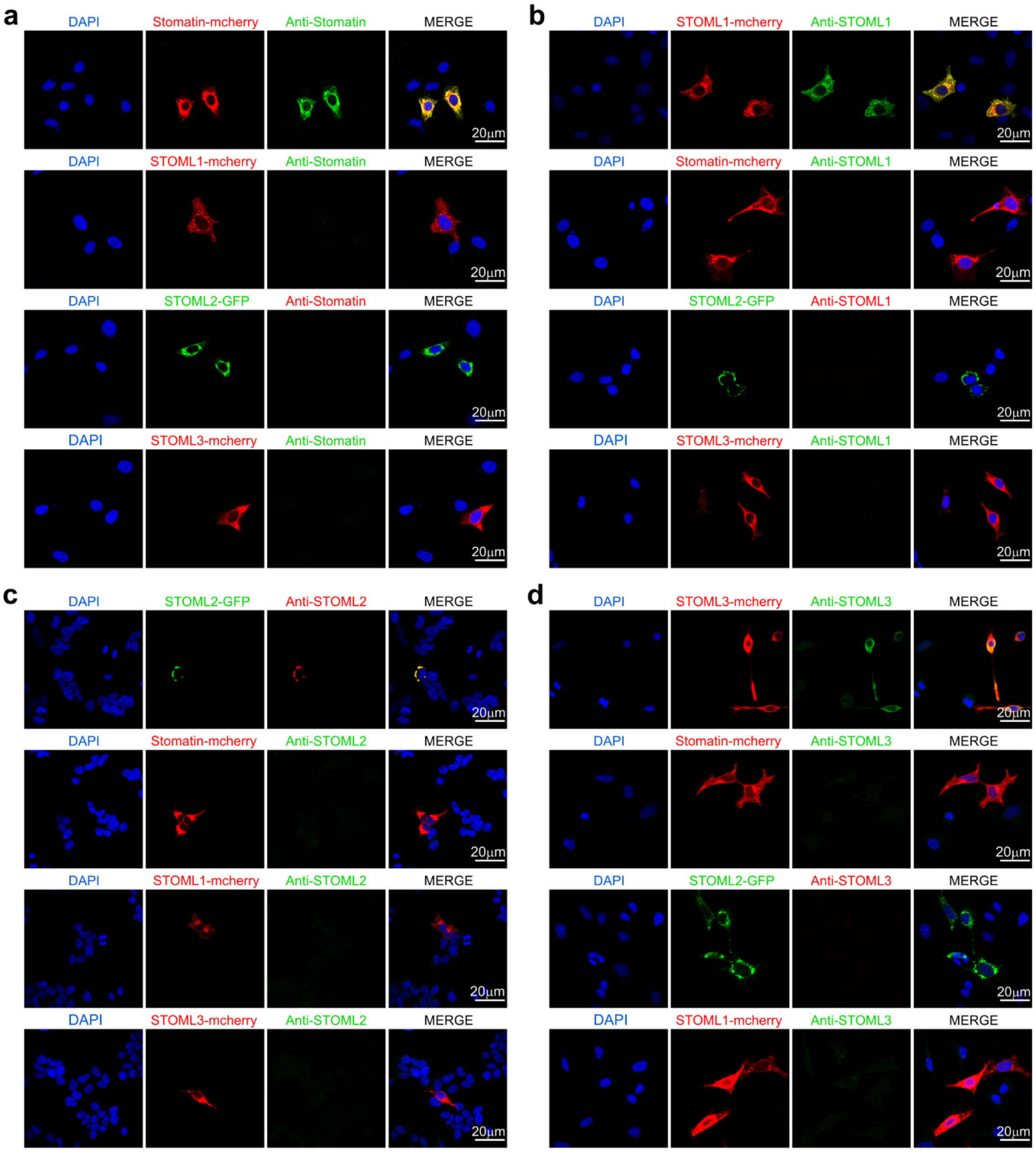

Supplementary figure 1. Antibodies to target stomatin-domain proteins are specific. Antibodies anti-STOM (**a**), anti-STOML1 (**b**), anti-STOML2 (**c**), and anti-STOML3 (**d**) were tested by immunocytochemistry in HEK-293 cells expressing the fusion proteins STOM-mCherry, STOML1-mCherry, STOML2-GFP, or STOML3-mCherry separately. The first column of each panel corresponds to the staining of the cell nuclei with DAPI in blue. The second panel corresponds to the signal coming from the fluorophore of the stomatin-domain protein/fluorophore fusion protein expressed heterologously. The third column of each panel corresponds to the signal obtained with each antibody using immunocytochemistry. The last column is the merge of the signals displayed in the previous three columns. Each of the antibodies specifically detected its respective target.

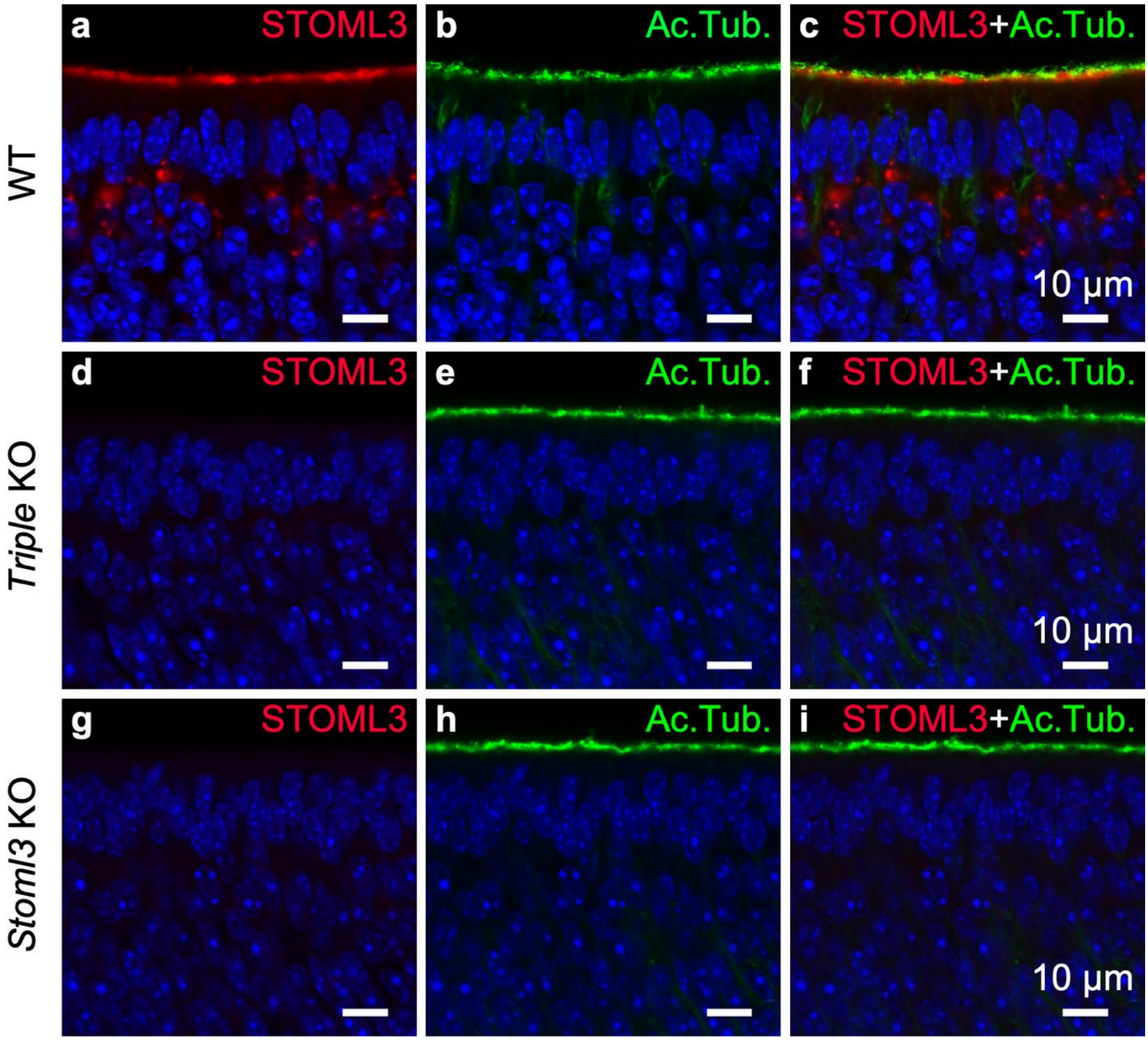

Supplementary figure 2. Acetylated tubulin and STOML3 staining in WT and KO mice. Single optical plane confocal micrographs of coronal sections of the OE of WT (**a-c**), *Triple* KO (**d-f**), and *Stoml3* KO (**g-i**) mice, double-stained with anti-STOML3 (red) and anti-acetylated tubulin (Ac. Tub., green) antibodies. Acetylated tubulin staining is similar in the three genotypes. STOML3 is localized in the distal part of the cilia and in the layer below acetylated tubulin in the region occupied by the OSN knobs (a-c). The signal in abolished in the OE of the *Triple* KO (**d-f**) and *Stoml3* KO mice (***g-i***). Nuclei were stained with DAPI (blue).

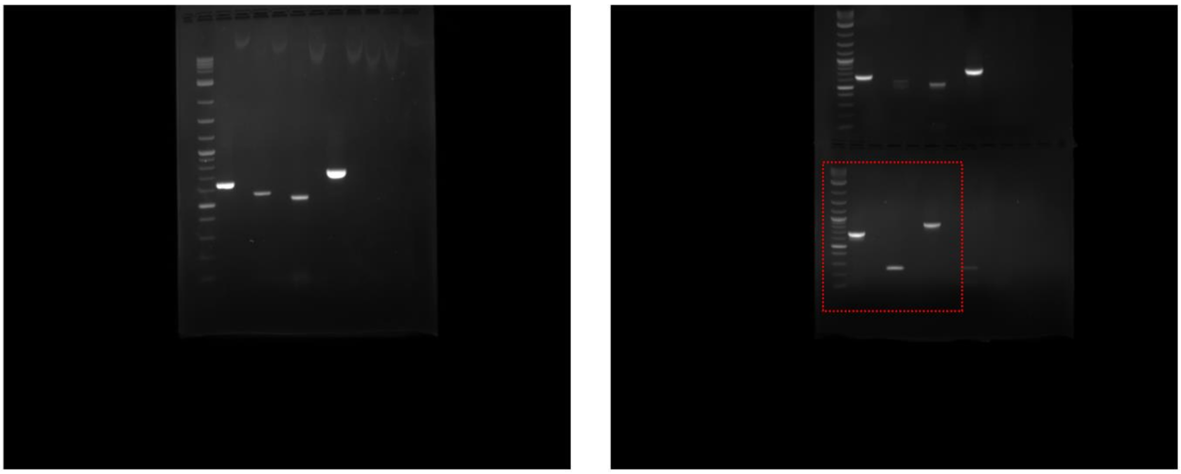

Supplementary figure 2. Original scanning of the gels shown in Figure 1
